# Hepatoblastoma: From Molecular Mechanisms to Therapeutic Strategies

**DOI:** 10.3390/curroncol32030149

**Published:** 2025-03-04

**Authors:** Ling Fan, Jintong Na, Tieliu Shi, Yuan Liao

**Affiliations:** 1 State Key Laboratory of Targeting Oncology, Guangxi Medical University, Nanning 530021, China; fanling@sr.gxmu.edu.cn (L.F.); najintong@sr.gxmu.edu.cn (J.N.); 2 National Center for International Research of Bio-Targeting Theranostics, Guangxi Medical University, Nanning 530021, China; 3 Guangxi Key Laboratory of Bio-Targeting Theranostics, Guangxi Medical University, Nanning 530021, China; 4 Collaborative Innovation Center for Targeting Tumor Diagnosis and Therapy, Guangxi Medical University, Nanning 530021, China; 5 Guangxi Talent Highland of Major New Drugs Innovation and Development, Guangxi Medical University, Nanning 530021, China; 6 Center for Bioinformatics and Computational Biology, Shanghai Key Laboratory of Regulatory Biology, The Institute of Biomedical Sciences and School of Life Sciences, East China Normal University, Shanghai 200241, China; 7 Key Laboratory of Advanced Theory and Application in Statistics and Data Science (MOE), School of Statistics, East China Normal University, Shanghai 200062, China

**Keywords:** hepatoblastoma, Wnt/β-catenin pathway, genetics, epigenetics, immunotherapy, targeted therapy

## Abstract

Hepatoblastoma (HB) is the most common malignant liver tumor in children under five years of age. Although globally rare, it accounts for a large proportion of liver cancer in children and has poor survival rates in high-risk and metastatic cases. This review discusses the molecular mechanisms, diagnostic methods, and therapeutic strategies of HB. Mutations in the CTNNB1 gene and the activation of the Wnt/β-catenin pathway are essential genetic factors. Furthermore, genetic syndromes like Beckwith–Wiedemann syndrome (BWS) and Familial Adenomatous Polyposis (FAP) considerably heighten the risk of associated conditions. Additionally, epigenetic mechanisms, such as DNA methylation and the influence of non-coding RNAs (ncRNAs), are pivotal drivers of tumor development. Diagnostics include serum biomarkers, immunohistochemistry (IHC), and imaging techniques. Standard treatments are chemotherapy, surgical resection, and liver transplantation (LT). Emerging therapies like immunotherapy and targeted treatments offer hope against chemotherapy resistance. Future research will prioritize personalized medicine, novel biomarkers, and molecular-targeted therapies to improve survival outcomes.

## 1. Introduction

Hepatoblastoma (HB) is the most prevalent malignant liver tumor in pediatric patients, primarily affecting those under five years of age [1]. Although rare globally, it constitutes nearly 70% of pediatric liver malignancies [1]. The exact etiology of HB remains unclear, but several risk factors have been identified, including prematurity, low birth weight, and genetic syndromes such as Beckwith–Wiedemann syndrome (BWS) and Familial Adenomatous Polyposis (FAP) [2]. Clinically, HB often presents with nonspecific symptoms like abdominal mass, jaundice, hepatomegaly, and failure to thrive [3,4]. In some cases, the tumor may be detected incidentally during imaging studies performed for other reasons. Despite significant advances in early diagnosis and treatment, survival rates for patients with advanced or recurrent disease remain suboptimal, particularly among high-risk populations. Children with metastatic disease or unresectable tumors face particularly poor outcomes, highlighting the urgent need for improved therapeutic strategies to enhance survival rates in these high-risk groups.

Molecularly, HB is characterized by genetic mutations and alterations in key signaling pathways that drive tumorigenesis. One of the most frequently altered pathways is the Wnt/β-catenin pathway, often activated by mutations in the CTNNB1 gene. These mutations disrupt the normal regulation of cell proliferation, differentiation, and apoptosis, driving tumor growth [5]. Emerging research also highlights the importance of epigenetic changes, including DNA methylation, histone modifications, and non-coding RNAs (ncRNAs), which influence gene expression and contribute to the aggressiveness of the tumor [2].

Current diagnostics leverage advanced imaging techniques and alpha-fetoprotein (AFP) for early detection and monitoring [6,7]. Treatment strategies usually combine surgical resection, chemotherapy, and liver transplantation (LT), especially for cases where surgery alone is not an option [8,9]. However, chemoresistance remains a significant challenge, driving ongoing research into novel therapies such as targeted treatments and immunotherapy to improve outcomes for patients with high-risk or advanced-stage HB [10].

This review provides a comprehensive overview of HB, focusing on recent advancements in its genetic and epigenetic basis, key signaling pathways, and diagnostic methods. It also evaluates current treatment strategies, addresses ongoing challenges, and explores future research directions aimed at improving survival outcomes and personalizing care for HB patients.

## 2. Molecular Mechanisms of Hepatoblastoma (HB)

### 2.1. Genetic Alterations

Genetic alterations play a crucial role in the development of HB. Understanding these changes is essential for improving diagnosis, treatment, and outcomes, particularly in children at high risk.

#### 2.1.1. Genetic Syndromes and Risk Factors for Hepatoblastoma (HB)

Several genetic syndromes are associated with an increased risk of HB due to specific genetic mutations. BWS is linked to changes in the 11p15.5 region, including the loss or gain of methylation and paternal uniparental disomy (UPD) [11]. These genetic alterations lead to the overexpression of insulin-like growth factor 2 (IGF2) [12], promoting excessive cell proliferation and increasing the risk of HB. CTNNB1 mutations, commonly observed in BWS-related HB, activate the Wnt/β-catenin pathway [13]. FAP is driven by mutations in the APC gene, leading to β-catenin activation. Unlike sporadic HB, FAP-related HB is primarily caused by APC mutations rather than CTNNB1 mutations [14]. The early detection of APC mutations is crucial for identifying individuals at risk before cancer develops [15]. Simpson–Golabi–Behmel syndrome (SGBS) results from mutations in the Glypican 3 (GPC3) gene, disrupting the Wnt/β-catenin pathway and promoting uncontrolled cell growth, which increases the risk of HB [16]. Somatic CTNNB1 mutations are also frequently found in HB cases in SGBS patients, highlighting the significant role of the Wnt/β-catenin pathway in HB development [17]. Trisomy 18 (Edwards syndrome) is associated with an increased risk of HB, although the exact mechanisms are unclear [18]. It is believed that the extra chromosome interferes with cell cycle regulation, promoting tumor formation [19]. AFP testing and ultrasound (US) screening are recommended for early detection in these patients [20]. Alagille syndrome (AGS), caused by mutations in the JAGGED1 or Notch2 genes, leads to liver dysfunction and cirrhosis, both of which elevate the risk of HB. A case report of a child with AGS and cirrhosis who developed HB emphasizes the importance of early screening for liver disease in AGS patients [21].

#### 2.1.2. Other Genetic Mutations in Hepatoblastoma (HB)

In addition to mutations associated with genetic syndromes, HB also exhibits somatic mutations in several key genes involved in processes such as differentiation, proliferation, and apoptosis. A major driver of HB is the CTNNB1 gene, which encodes β-catenin, a crucial component of the Wnt/β-catenin signaling pathway. Mutations in CTNNB1, often leading to its constitutive activation, are found in a significant proportion of HB cases and are closely associated with the fetal or embryonal subtype of the disease [22]. Additionally, AXIN2 and PARP1 have been identified as additional risk genes. Mutations in AXIN2 disrupt the Wnt signaling pathway, potentially contributing to tumorigenesis in HB [23]. Beyond these, BRCA2 and GPC3 are emerging as novel candidate genes associated with HB. BRCA2 mutations, often linked to DNA repair defects, may influence tumor progression, while GPC3, involved in cell signaling and adhesion, may play a role in HB biology, although its precise contribution requires further validation [23]. Moreover, specific mutations, such as TERT promoter mutations, commonly found in older patients, have been associated with poor prognosis and advanced disease stages [24]. These findings highlight the complex and heterogeneous nature of HB, with genetic alterations influencing various aspects of tumor biology, including treatment response and overall prognosis.

### 2.2. Epigenetic Alterations

Epigenetic modifications include DNA methylation, histone modifications, and ncRNAs. They regulate gene expression and cellular processes that drive HB development and progression. These changes impact critical signaling pathways. They influence cell proliferation, differentiation, and apoptosis. Understanding these mechanisms provides valuable insights into how this aggressive cancer grows and spreads.

#### 2.2.1. DNA Methylation

Recent studies have demonstrated that DNA methylation plays a significant role in the pathogenesis of HB, influencing both tumor progression and prognosis. One of the most prominent features of HB is its global hypomethylation pattern, accompanied by the hypermethylation of specific tumor suppressor genes (TSGs). Genes such as RASSF1A, SOCS1, APC, and P16 are frequently silenced in HB cells due to DNA promoter methylation, disrupting critical cellular pathways. In particular, the WNT/β-catenin pathway, which is crucial for liver development, is significantly altered in HB [25]. The downregulation of SFRP1, a key antagonist of the WNT signaling pathway, due to DNA methylation has been correlated with β-catenin mutations, highlighting the interplay between epigenetic modifications and genetic alterations in HB [26].

Furthermore, DNA methylation profiling has contributed to refining risk stratification models for HB. Recent studies have identified distinct epigenomic clusters, including Epi-CA and Epi-CB. The Epi-CB cluster, characterized by strong 14q32 locus expression and DNA hypomethylation, is associated with a more aggressive clinical phenotype and mutations in genes such as CTNNB1 and NFE2L2, as well as a progenitor-like cellular phenotype [27]. Additionally, DNA methylation alterations have been shown to correlate with poor clinical outcomes, including reduced overall survival (OS) and event-free survival (EFS). The methylation of genes such as RASSF1A, PARP6, OCIAD2, and MST1R has been linked to worse prognosis [28]. Oncogenes such as IGF2 also exhibit the hypomethylation of their fetal promoter, leading to their overexpression, which correlates with more aggressive tumor features, including a progenitor-like phenotype and shorter recurrence-free survival [29]. These findings suggest that DNA methylation not only contributes to tumorigenesis but also serves as a crucial biomarker for predicting clinical outcomes in HB patients.

The growing understanding of DNA methylation alterations in HB has paved the way for novel therapeutic strategies. Targeting epigenetic regulators such as G9a and UHRF1 have shown promising results in preclinical models. G9a, a histone methyltransferase, has been identified as a potential therapeutic target, with its inhibition in HB cell lines and animal models demonstrating significant antitumor effects, providing hope for improved treatment outcomes. Similarly, the dual inhibition of UHRF1 and DNMT1, in combination with conventional therapies like cisplatin (CDDP), has shown encouraging results, particularly for high-risk HB patients [30]. Furthermore, integrating DNA methylation analysis into existing clinical risk stratification models, such as the Children’s Hepatic Tumors International Collaboration-HB Stratification (CHIC-HS), has enhanced prognostic accuracy and personalized treatment strategies [28]. These advancements underscore the potential of DNA methylation profiling to improve patient management and guide the development of more targeted therapies for HB.

#### 2.2.2. Histone Modifications

Histone modifications, including acetylation, methylation, and phosphorylation, play a crucial role in HB by regulating chromatin structure and gene expression. Dysregulated histone modifications can lead to the silencing of TSGs and the activation of oncogenes. For instance, the overexpression of UHRF1 silences critical TSGs such as HHIP and IGFBP3 through histone modifications and DNA methylation. This overexpression is associated with poor survival rates [31]. Similarly, the histone methyltransferase G9a is upregulated in HB, promoting tumor growth by modifying histones and regulating key pathways such as Wnt/β-catenin [32].

Histone modifications in HB are also linked to metabolic reprogramming and chemotherapy resistance. Sun et al. [33] demonstrated that metabolic changes in cancer cells are associated with histone modifications, contributing to tumorigenesis. Targeting histone-modifying enzymes like G9a has shown promising therapeutic potential. The inhibition of G9a reduced tumor growth and improved chemotherapy efficacy in HB models, suggesting that epigenetic modulation could enhance treatment strategies. Overall, histone modifications are pivotal in HB progression, and targeting these modifications may lead to more effective therapies for this pediatric cancer.

#### 2.2.3. Non-Coding RNAs (ncRNAs)

NcRNAs, including long non-coding RNAs (lncRNAs) and microRNAs (miRNAs), have emerged as vital regulators in HB pathogenesis. LncRNAs, defined as transcripts longer than 200 nucleotides, regulate various aspects of HB biology. For example, Linc00205 is upregulated in HB tissues and promotes tumor development by sponging miR-154-3p, thereby activating the MAPK pathway and increasing ROCK1 expression [34]. Similarly, LncRNA MIR205HG activates the MAPK and PI3K/AKT pathways, promoting tumor cell proliferation, migration, and invasion [35]. Linc01023 regulates the miR-378a-5p/WNT3 axis, enhancing tumorigenesis by promoting cell proliferation and colony formation [36]. Additionally, HAND2-AS1 functions as a tumor suppressor in HB by inhibiting CDK1, thus suppressing cell proliferation and progression through the cell cycle [37]. Exosomal lncRNA NEAT1 has been associated with HB progression by inducing bone marrow stromal cell (BMSC) differentiation into tumor-promoting myofibroblasts through the miR-132/MMP9 axis [38].

MiRNAs, small ncRNAs approximately 22 nucleotides in length, regulate gene expression by binding to the 3′ untranslated regions (3′ UTRs) of target mRNAs. Dysregulated miRNA expression is a hallmark of various cancers, including HB. For instance, miR-135a inhibits tumor cell proliferation by modulating the Notch pathway, a critical regulator of the cell cycle and differentiation [39]. Wu et al. [40] found that the miR-139-3p/Wnt5A axis inhibits HB metastasis, with the overexpression of miR-139-3p reducing HB cell invasion and migration.

Both lncRNAs and miRNAs serve crucial roles in regulating the signaling pathways involved in HB tumorigenesis. LncRNAs like Linc00205, MIR205HG, and NEAT1, along with miRNAs like miR-135a and miR-139-3p, influence key processes such as cell proliferation, migration, invasion, and metastasis by regulating pathways like MAPK, PI3K/AKT, and Notch. These findings underscore the potential of ncRNAs as biomarkers for diagnosis and prognosis in HB and suggest that targeting these molecules holds promise for novel therapeutic strategies aimed at improving HB treatment outcomes. Table 1 presents the role and mechanisms of lncRNAs and miRNAs in HB.

#### 2.2.4. Single-Cell Sequencing and Epigenetics

Epigenetic regulation plays a crucial role in tumor heterogeneity in HB, shaping the diversity of tumor cell populations and their responses to treatment. Recent advances in single-cell sequencing have provided unprecedented insights into the molecular mechanisms that drive HB tumorigenesis and progression. A key feature of this heterogeneity is the presence of distinct subpopulations of tumor cells with varying differentiation states, such as hepatocytic, liver progenitor, and mesenchymal-like cells. These subpopulations exhibit different epigenetic profiles, which influence their proliferation, differentiation, and chemotherapy resistance, contributing to overall tumor plasticity [41]. Single-cell transcriptomics has revealed that the gene regulatory networks governing these subpopulations are influenced by chromatin accessibility and DNA methylation patterns, which regulate their differentiation and proliferative capacity.

Studies have shown that DNA methylation and histone modifications are integral to tumor heterogeneity in HB. Profiling at the single-cell level has identified the overexpression of genes related to stem cell properties and DNA repair in progenitor-like subpopulations, especially those that proliferate rapidly after chemotherapy [41,42]. These epigenetic alterations not only initiate tumorigenesis but also help tumor cells adapt to therapy, contributing to chemoresistance and poor prognosis. Additionally, metabolic pathways linked to changes in the epigenetic landscape reinforce the heterogeneity observed in HB tumors [43]. By mapping somatic alterations and chromatin landscapes at single-cell resolution, researchers have uncovered that distinct genetic subclones exhibit varying levels of cellular plasticity, which influences their ability to transition between differentiation states and respond to chemotherapy [41]. These insights into the dynamic regulation of HB tumor heterogeneity emphasize the potential for precision medicine strategies targeting specific epigenetic alterations. For example, inhibiting epigenetic regulators like FACT could offer a promising treatment approach for high-risk HB subtypes exhibiting stem-like properties [44]. These findings underscore the importance of combining single-cell sequencing with epigenetic profiling to develop personalized treatment strategies for this aggressive pediatric cancer.

### 2.3. Signaling Pathways

HB is an aggressive liver tumor characterized by dysregulated signaling pathways that drive tumor growth, metastasis, and chemoresistance. Key signaling pathways implicated in the pathogenesis of HB include Wnt/β-catenin, Hippo, Notch, transforming growth factor-beta (TGF-β), insulin-like growth factor (IGF), PI3K/Akt/mTOR, MAPK/ERK, and hepatocyte growth factor (HGF)/c-Met. These pathways interact within complex networks that amplify the malignant transformation of liver cells, ultimately contributing to poor prognosis in HB patients.

#### 2.3.1. Wnt/β-Catenin Pathway

The activation of the Wnt/β-catenin signaling pathway is mediated by Wnt proteins, a family of secreted glycoproteins. Wnt ligands bind to Frizzled (FZD) receptors on the cell membrane and to the co-receptor lipoprotein receptor-related proteins 5 or 6 (LRP 5/6), initiating the downstream activation of the Wnt/β-catenin pathway [45,46]. This binding recruits the scaffolding protein Disheveled (Dvl), leading to the phosphorylation of LRP5/6 and Axin at the plasma membrane [47]. The recruitment of these proteins disrupts the β-catenin destruction complex, resulting in the stabilization and accumulation of β-catenin in the cytoplasm. β-catenin then translocates to the nucleus, where it forms a complex with T cell factor/lymphoid enhancer factor (TCF/LEF) transcription factors, driving the expression of Wnt target genes. In the absence of Wnt proteins, β-catenin is kept at low levels through degradation by the destruction complex, which consists of Axin, adenomatous polyposis coli (APC), glycogen synthase kinase 3 beta (GSK3β), and casein kinase 1 alpha (CK1α).

The Wnt/β-catenin pathway plays a central role in HB development and is frequently activated by mutations in the CTNNB1 gene, which inhibit the proteasomal degradation of β-catenin. As a result, β-catenin accumulates in the nucleus, activating oncogenic genes and promoting tumor progression. CTNNB1 mutations, particularly in exon 3, are found in approximately 90% of HB cases [48]. Nuclear β-catenin localization is linked to more aggressive tumor subtypes and poor prognosis [49]. While the Wnt/β-catenin pathway is essential for liver development and regeneration, its dysregulation drives tumor formation, making it a promising therapeutic target. SOX7, a tumor suppressor, is downregulated in HB tissues, whereas β-catenin is upregulated. Restoring SOX7 expression inhibits Wnt/β-catenin signaling, reducing tumor growth and invasion [50]. Other factors also influence this pathway. Fascin-1, a protein linked to tumor differentiation, is upregulated in HB due to β-catenin mutations and is associated with poor prognosis [51]. Additionally, KDM1A, a lysine demethylase, exacerbates HB progression by inhibiting DKK3, a negative regulator of the Wnt/β-catenin pathway [52].

Therapies targeting this pathway show promise. Aprepitant, a small molecule, inhibits Wnt/β-catenin signaling, thereby reducing tumor cell proliferation and invasiveness [53]. Another potential therapy, quercetin, activates the tumor suppressor SIRT6, which inhibits FZD4 and suppresses Wnt/β-catenin signaling [54]. Targeting the Wnt/β-catenin pathway offers a promising approach to improve outcomes for HB patients, particularly those with aggressive or recurrent tumors. These strategies are contributing to the development of more effective treatments.

#### 2.3.2. Hippo Pathway

The Hippo signaling pathway is an important survival-related pathway, and its inactivation can increase cell proliferation and reduce apoptosis, leading to tumorigenesis and progression. Yes-associated protein 1 (YAP)/transcription regulator 1 (TAZ) are typically identified as oncogenes, while mammalian STE20-like kinase 1/2 (MST1/2) and large tumor suppressor kinase 1/2 (LATS1/2) are recognized as tumor suppressors. The upstream striatin (STRN)-interacting phosphatase and kinase (STRIPAK) complex of the Hippo pathway regulates MST1/2 and MAP4K. MAP4K or MST1/2 and their scaffold protein salvador homolog 1 (SAV1) can phosphorylate LATS1/2 and its scaffold MOBKL1 (MOB1) with the help of WWC1-3. Phosphorylated MOB1 can also promote LATS1/2 activation by inducing conformational changes in LATS1/2. When the Hippo pathway is activated, the activity of YAP/TAZ is inhibited by LATS1/2-mediated phosphorylation. When the Hippo pathway is inactivated, dephosphorylated YAP/TAZ translocates to the nucleus and binds with the transcription factor transcriptional enhanced associated domain1-4 (TEAD1-4) to induce gene expression [55].

The primary effect of the Hippo pathway is mediated through the activation of YAP. In HB, the inactivation of LATS leads to YAP activation. Activated YAP accumulates in the nucleus and drives the expression of genes that promote cell survival, proliferation, and metastasis [56]. Elevated YAP activity is associated with aggressive tumor features, such as vascular invasion, lymph node metastasis, and poor prognosis [57]. YAP also promotes angiogenesis by inducing the expression of vascular endothelial growth factor (VEGF), which is essential for tumor blood supply. Furthermore, YAP interacts with β-catenin, a key protein in the Wnt signaling pathway, fostering tumor growth and sustaining cancer stem cell (CSC) populations [58]. The cooperation between YAP and β-catenin significantly accelerates HB progression, making it a key factor in the disease.

In addition, TAZ, another important effector of the Hippo pathway, interacts with β-catenin to further drive tumor progression. In mouse models, the co-overexpression of active TAZ (TAZS89A) and ΔN90-β-catenin has been shown to induce HB lesions, highlighting the crucial role of the TAZ/β-catenin interaction in HB development [59]. Moreover, the N6-methyladenosine (m6A) modification of LATS2, a critical component of the Hippo pathway, has been implicated in tumor progression by suppressing ferroptosis through the YAP1/ATF4/PSAT1 axis. Targeting the restoration of the LATS2 function or inhibiting YAP activity may offer promising therapeutic avenues for HB treatment [60].

#### 2.3.3. Notch Pathway

The Notch signaling pathway comprises four receptors (Notch1-4) and five ligands: Jagged1 and Jagged2 (JAG1 and JAG2), as well as Delta-like ligands (DLL1, DLL3, and DLL4). When the extracellular domain of a notch receptor (NECD) interacts with a ligand on an adjacent cell, the receptor is activated. This interaction induces a conformational change in the notch receptor, exposing its cleavage site. A series of proteolytic events follows: The ADAM metalloprotease cleaves the S2 site, generating the notch extracellular truncation (NEXT) intermediate. Two additional cleavages occur at the S3 and S4 sites of the transmembrane domain by γ-secretase, releasing the notch intracellular domain (NICD). The NICD then rapidly translocates to the nucleus, where it associates with the CSL and Mastermind-like (MAML) transcriptional complex to regulate the transcriptional activity of notch target genes [61].

Notch signaling plays a crucial role in regulating cell fate decisions during liver development, and its dysregulation contributes to HB progression. Notch2, a key receptor in the notch family, is overexpressed in 92% of HB cases compared to normal liver tissue, highlighting its role in maintaining an undifferentiated hepatoblast state. The sustained activation of Notch2 inhibits differentiation, leading to uncontrolled proliferation and tumor progression. Interestingly, Notch2 activation in HB occurs independently of its ligand, JAGGED1, suggesting a unique regulatory mechanism in HB [62].

Notch signaling also interacts with the Wnt/β-catenin pathway to coordinate hepatoblast differentiation. López-Terrada et al. [63] reported that notch activation is predominantly observed in HB subtypes with fetal features, while Wnt/β-catenin activation is more common in embryonal and mixed types, contributing to the molecular heterogeneity and clinical variability of HB.

**Figure 1:**
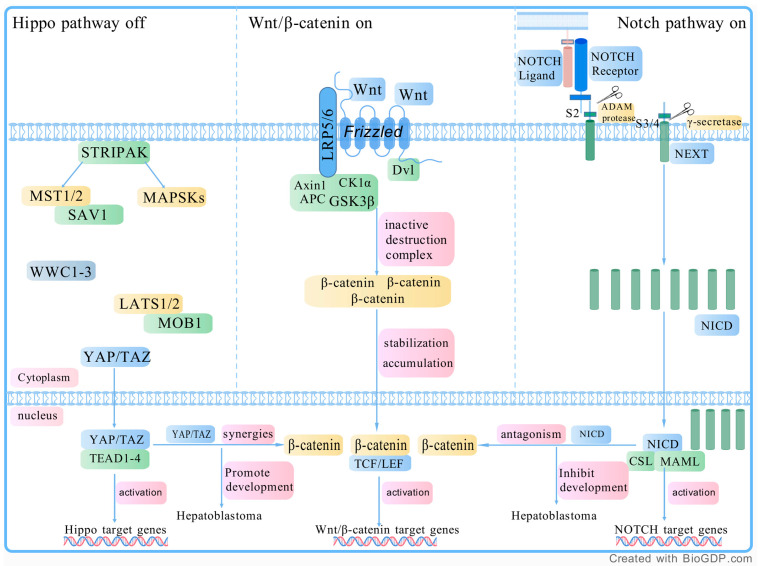
The interaction of the WNT pathway, Hippo pathway, and Notch pathway. Wnt pathway: The Wnt/β-catenin signaling pathway is activated when Wnt proteins bind to Frizzled (FZD) receptors and the co-receptor LRP5/6 on the cell membrane. This binding recruits the scaffolding protein Disheveled (Dvl), which leads to the phosphorylation of LRP5/6 and Axin. This disrupts the β-catenin destruction complex, allowing β-catenin to accumulate in the cytoplasm. Once stabilized, β-catenin moves into the nucleus, where it forms a complex with TCF/LEF transcription factors to activate the expression of Wnt target genes. In the absence of Wnt proteins, β-catenin is degraded by the destruction complex, which includes Axin, APC, GSK3β, and CK1α, keeping its levels low. Hippo pathway: The upstream STRIPAK complex regulates MST1/2 and MAP4K, which, along with the scaffold protein SAV1, phosphorylate LATS1/2 and the scaffold protein MOB1. The phosphorylation of MOB1 promotes LATS1/2 activation by inducing conformational changes. Additionally, the WWC1-3 proteins support this process by assisting in the phosphorylation of both LATS1/2 and MOB1. When the Hippo pathway is activated, LATS1/2 phosphorylates and inhibits YAP/TAZ. However, when the Hippo pathway is inactivated, dephosphorylated YAP/TAZ translocates to the nucleus, where it binds to TEAD transcription factors, thereby inducing gene expression that can drive tumor growth. Notch pathway: When the NECD binds to a ligand on an adjacent cell, it activates the receptor, causing a conformational change that exposes a cleavage site. First, the ADAM metalloprotease cleaves the S2 site, producing a notch intermediate called NEXT. Then, γ-secretase cleaves the S3 and S4 sites in the transmembrane domain, releasing the NICD. NICD then translocates to the nucleus, where it forms a complex with CSL and MAML, regulating the expression of notch target genes. Abbreviations: LRP 5/6, lipoprotein receptor-related proteins 5 or 6; TCF/LEF, T cell factor/lymphoid enhancer factor; APC, adenomatous polyposis coli; GSK3β, glycogen synthase kinase 3 beta; CK1α, casein kinase 1 alpha; YAP, yes-associated protein 1; TAZ, transcription regulator 1; MST1/2, mammalian STE20-like kinase 1/2; STRIPAK, striatin (STRN)-interacting phosphatase and kinase; SAV1, salvador homolog 1; MOB1, MOBKL1; TEAD1-4, transcriptional enhanced associated domain1-4; NECD, notch extracellular domain; NEXT, notch extracellular truncation; NICD, notch intracellular domain; NECD, notch extracellular domain; MAML, Mastermind-like. Created with BioGDP.com (accessed on 19 February 2025).

The Wnt, Hippo, and Notch pathways form a crosstalk network that influences each other, collectively promoting tumor growth and proliferation. The synergistic interaction between YAP/TAZ and β-catenin accelerates HB progression [59]. A study by Acar et al. demonstrated that the NICD directly interacts with β-catenin and suppresses its transcriptional activity to promote cell differentiation. This interaction drives a Notch-ON/Wnt-OFF state, which in turn impacts HB progression [64]. Figure 1 illustrates the interactions among the Wnt, Hippo, and Notch pathways.

#### 2.3.4. TGF-β Pathway

TGF-β activates Smad proteins, particularly Smad2 and Smad3, through receptor-mediated phosphorylation. These Smads then form heteromeric complexes with Smad4, which regulate the expression of target genes in the nucleus [65]. The dysregulation of this pathway in HB disrupts cell cycle control and apoptosis, contributing to tumorigenesis. TGF-β1 is closely associated with HB growth and metastasis. Buenemann et al. [65] found that while TGF-β induces growth arrest and apoptosis in some liver cancer cells, HB cells such as HepG2 exhibit resistance, allowing continued proliferation. Moreover, TGF-β promotes epithelial–mesenchymal transition (EMT), which facilitates metastasis. Fu et al. [66] demonstrated that thymosin β4 (Tβ4) induces EMT, driving HB metastasis.

TGF-β also interacts with other signaling pathways in HB. Matsumoto et al. [67] showed that Wnt/β-catenin signaling suppresses TGF-β by upregulating GREB1, which inhibits Smad2/3 activity, thereby promoting HB proliferation. This interaction underscores the complexity of HB and suggests the potential for dual-target therapies. Additionally, TGF-β contributes to chemoresistance. Xiang et al. [68] identified a TGF-β-driven chemoresistant phenotype in the S2A subtype, which is linked to a pro-fibrotic and immunosuppressive microenvironment. This results in poor prognosis and highlights the need for targeted strategies to inhibit TGF-β.

#### 2.3.5. IGF Pathway

Key components of the IGF axis, including IGF2, IGF1R, and IGFBP3, are frequently dysregulated in HB, promoting tumor development and metastasis. Gray et al. [69] demonstrated that alterations in IGF axis members are crucial for HB tumorigenesis. IGF2, an essential growth factor for fetal liver development, is overexpressed in HB, often accompanied by a loss of imprinting at the IGF2/H19 imprinted gene locus [70]. This overexpression activates IGF1R, which in turn triggers downstream PI3K-Akt and MAPK pathways, driving cell proliferation in HB [71]. Further studies have revealed the complex regulation of the IGF axis in HB. Regel et al. [70] found that IGFBP3, an inhibitor of IGF2, is epigenetically silenced in metastatic HB, facilitating aggressive behavior. Additionally, Zhen et al. [72] identified circHMGCS1 as a key regulator of IGF signaling in HB, which sponges miR-503-5p, enhancing IGF2 and IGF1R expression and activating the PI3K-Akt pathway. These findings highlight the interplay between the IGF axis and other pathways like PI3K-Akt and Wnt/β-catenin in HB progression. Targeting IGF2, IGF1R, and their downstream signaling components offers potential therapies for aggressive or metastatic HB.

#### 2.3.6. PI3K/AKT/mTOR Pathway

The dysregulation of the PI3K/AKT/mTOR pathway significantly contributes to HB progression. Liu et al. [73] found that mTORC1, a key downstream effector of PI3K/Akt, is activated in HB and is essential for tumor development. The inhibition of mTORC1 reduced HB cell growth, underscoring its role in progression. Similarly, Cui et al. [74] demonstrated that Dipeptidase 1 (DPEP1) promotes HB progression via the PI3K/Akt/mTOR pathway. Silencing DPEP1 suppressed HB cell proliferation and migration, while its overexpression had the opposite effect. Barros et al. [75] linked PI3K/Akt signaling to copy number alterations (CNAs) in HB, showing how these genetic changes activate the pathway and contribute to aggressiveness. The PI3K/Akt/mTOR pathway also interacts with Wnt/β-catenin signaling. Berkemeyer [76] demonstrated that cross-talk between these pathways synergistically promotes tumor progression. Targeting PI3K or mTOR represents a promising therapeutic approach for managing aggressive HB.

#### 2.3.7. MAPK/ERK Pathway

The MAPK/ERK pathway plays a crucial role in HB progression by regulating cell migration, invasion, and metastasis. Chen et al. [77] showed that periostin (POSTN), an extracellular matrix protein, promotes EMT and HB progression by activating MAPK/ERK signaling. POSTN upregulates Snail and downregulates OVOL2, enhancing migration and invasion. This suggests that the POSTN-mediated activation of MAPK/ERK could be a potential therapeutic target. Li et al. [78] found that LASP2, a cytoskeletal protein, is downregulated in HB, and its loss promotes malignant phenotypes through MAPK/ERK signaling. Silencing LASP2 increased ERK and p-ERK expression, which in turn enhanced proliferation and migration, while inhibiting ERK reversed these effects. Chung et al. [79] demonstrated that GPC3-deficient HB cells exhibit the upregulation of MAPK/ERK signaling, leading to reduced tumorigenicity and increased sensitivity to ERK inhibitors. These findings emphasize the importance of MAPK/ERK signaling in HB, particularly in aggressive forms of the disease.

#### 2.3.8. HGF/c-Met Pathway

The aberrant activation of the HGF/c-Met pathway is frequently observed in HB and is associated with its invasive behavior. Purcell et al. [80] found that c-Met activation leads to the phosphorylation of β-catenin, a key regulator in HB, promoting tumor progression. Their study demonstrated elevated cytoplasmic and nuclear levels of phosphorylated β-catenin in HB samples, underscoring the critical role of c-Met in activating β-catenin and driving HB malignancy.

Further research has revealed additional interactions within this pathway. Cui et al. [81] showed that the lncRNA ZFAS1 influences HB growth by sponging miR-193a-3p and targeting RALY, a key component of the HGF/c-Met pathway. Elevated ZFAS1 expression correlates with more aggressive tumor features and poorer survival outcomes. Additionally, Zhang et al. [82] demonstrated that HGF/c-Met signaling interacts with other pathways, such as Wnt/β-catenin, to enhance tumor growth. These interactions suggest that the HGF/c-Met axis plays a pivotal role in HB progression. Targeting the HGF/c-Met pathway offers promising therapeutic opportunities, as it may help inhibit HB growth and metastasis, ultimately improving outcomes for patients with aggressive disease.

The development of HB is driven by intricate interactions among various signaling pathways that regulate critical processes in tumor progression. These pathways form a robust network that sustains tumor growth and complicates treatment, highlighting the need for targeted therapies and combination strategies to improve patient outcomes. Table 2 summarizes the signaling pathways involved in HB and their regulatory roles in tumorigenesis.

### 2.4. Alterations in Metabolism Pathways

HB is characterized by significant metabolic reprogramming, which plays a crucial role in tumor growth and progression. One of the most prominent metabolic shifts observed in HB is the enhancement of glycolysis, commonly known as the Warburg effect. This shift allows HB cells to generate energy rapidly, even in the presence of oxygen, a characteristic commonly seen in many cancer cells. The Warburg effect in HB is further supported by brain-expressed X-linked protein 1 (BEX1), a protein that promotes stemness in HB cells by modulating the peroxisome proliferator-activated receptor gamma (PPARγ) and pyruvate dehydrogenase kinase isozyme 1 (PDK1) axis. This metabolic adaptation not only sustains cell proliferation but also contributes to a poor prognosis and resistance to chemotherapy [83].

In addition to alterations in glycolysis, HB also exhibits significant changes in fatty acid metabolism. A key feature of this reprogramming is the suppression of mitochondrial fatty acid β-oxidation, particularly due to the downregulation of enzymes such as CPT1a. This results in a reduced utilization of long-chain fatty acids, which in turn leads to the accumulation of branched-chain amino acids and disturbances in carnitine metabolism. These changes reduce oxidative phosphorylation and contribute to mitochondrial dysfunction, further promoting the aggressive nature of HB and creating an environment that supports rapid tumor growth [84].

Furthermore, altered enzymes like DNMT3B and PFKFB4 have been linked to metastatic traits in HB, suggesting that metabolic changes may also facilitate tumor spread and metastasis [42]. Additionally, mitochondrial dysfunction, driven by c-Myc overexpression, causes mitochondrial fragmentation, which increases the production of reactive oxygen species (ROS) and activates oncogenic signaling pathways such as AKT/mTOR and NF-κB. These metabolic shifts collectively promote tumorigenesis, survival, and resistance to therapeutic interventions, emphasizing their potential as therapeutic targets for HB treatment [85].

### 2.5. The Tumor Microenvironment

The tumor microenvironment (TME) of HB plays a crucial role in its progression and resistance to therapy. One key feature of the HB TME is immune cell infiltration and the mechanisms of immune evasion. Natural killer (NK) cells, although present in higher numbers in HB tissues compared to normal liver tissues, have their cytotoxic activity inhibited by the interaction between HLA-C on tumor cells and KIR2DL receptors on NK cells. This interaction allows the tumor to evade immune detection, fostering tumor growth despite the presence of immune cells [86]. Additionally, tumor-associated macrophages (TAMs) contribute significantly to HB tumor progression by promoting an immunosuppressive environment that supports tumor proliferation, invasion, and chemoresistance. The interaction between TAMs and HB cells forms feedback loops, enhancing the tumor’s aggressiveness [87].

In addition to immune evasion, angiogenesis plays a critical role in HB. Angiogenesis, the formation of new blood vessels, is essential for supplying nutrients and oxygen to the growing tumor. Extracellular matrix (ECM) remodeling and VEGF contribute to the angiogenic network that supports tumor growth and metastasis [88]. Stromal components, including fibroblasts and cancer-associated fibroblasts (CAFs), secrete pro-angiogenic factors that promote the formation of new blood vessels and enhance tumor progression. Furthermore, the interaction between tumor cells and stromal cells is essential for shaping the TME. Tumor cells in HB reprogram macrophages within the TME into an immunosuppressive state, further promoting tumor progression and immune evasion [89].

Moreover, Wnt/β-catenin signaling, frequently activated due to CTNNB1 mutations in HB, significantly influences the TME by promoting immune evasion. This pathway upregulates midkine, an immunomodulator that alters macrophage phenotypes and contributes to immune exclusion from tumor areas [89]. The resulting immune exclusion diminishes the efficacy of immune responses, complicating treatment strategies, particularly immunotherapies. Overall, the immune microenvironment, angiogenesis, and stromal cell-tumor cell interactions collectively contribute to the aggressive nature and therapy resistance of HB, highlighting potential therapeutic targets such as midkine inhibition and TAM reprogramming [88,90].

## 3. Diagnostic Approaches for Hepatoblastoma (HB)

The accurate diagnosis of HB requires a comprehensive approach. Current strategies include serum markers, immunohistochemistry (IHC), and advanced imaging techniques. Early detection is critical for better outcomes. These methods help in diagnosis, staging, and treatment planning. They guide therapy decisions, track disease progression, and evaluate treatment response. A precise diagnostic approach is key to improving patient care.

### 3.1. Serum Markers

Serum biomarkers play a crucial role in the diagnosis and management of HB. Among these, AFP is the most established biomarker, with its AFP-L3 fraction proving especially useful for predicting recurrence. In a study by Kawahara et al. [91], while AFP levels did not show significant differences in the early postoperative period, AFP-L3 levels fell below the detection limit in 70% of patients in the non-recurrence group but in none of the recurrence group. This finding suggests that AFP-L3 could be a more sensitive and earlier predictor of HB recurrence following surgery. Additionally, circulating tumor DNA (ctDNA), specifically mutations in CTNNB1, has demonstrated considerable diagnostic potential. Kahana-Edwin et al. [92] used their bespoke QUENCH sequencing assay to detect ctDNA in plasma samples from HB patients, achieving a sensitivity of 90% at initial diagnosis. They found that ctDNA levels correlated with tumor burden, macroscopic residual disease, and treatment response, underscoring its utility as a non-invasive tool for monitoring HB progression alongside traditional markers like AFP.

In addition to these well-established biomarkers, serum uric acid (SUA) has been identified as a promising prognostic marker. Zhou et al. [93] observed that elevated SUA levels were associated with advanced tumor staging (PRETEXT stage IV), vascular involvement, and multifocality at diagnosis and remained elevated in patients with poor treatment responses following neoadjuvant chemotherapy. Importantly, higher post-treatment SUA levels were linked to worse 5-year EFS and OS, suggesting SUA’s potential to assess tumor staging and predict clinical outcomes. When combined, AFP, ctDNA, and SUA form a robust diagnostic framework, enhancing the ability to stratify risk, monitor disease progression, and evaluate treatment efficacy in HB, offering clinicians a comprehensive approach to patient management

### 3.2. Immunohistochemical Markers

Immunohistochemical (IHC) markers can predict the prognosis of HB and help to distinguish it from other pediatric liver tumors and assess its molecular features. Among the key IHC markers, β-catenin is central to HB pathogenesis due to its involvement in the Wnt/β-catenin signaling pathway. The intracellular localization of β-catenin, whether nuclear, cytoplasmic, or membranous, provides valuable prognostic insights. Nuclear β-catenin expression, particularly in embryonal/undifferentiated HB, is strongly associated with poor chemotherapy response, increased vascular involvement, and reduced survival [49]. Conversely, cytoplasmic β-catenin expression has been linked to a more aggressive disease course, further emphasizing its importance in determining treatment outcomes [94]. Glutamine synthetase (GS), another crucial IHC marker, is often expressed in the epithelial component of untreated HB and is useful for assessing tumor differentiation, especially post-chemotherapy. GS immunostaining is particularly beneficial for evaluating surgical margins after neoadjuvant chemotherapy, helping detect residual disease and refine therapeutic strategies [95].

The emerging marker CD203c, found on fetal hepatoblasts and some embryonal HB components, offers additional diagnostic value. Its expression correlates with the less differentiated and more aggressive tumor phenotype, providing a potential tool for identifying subsets of HB. CD203c may also play a role in purinergic signaling during liver development, further shedding light on HB’s molecular pathways [96]. When combined with β-catenin and GS, CD203c enhances the ability to assess HB’s differentiation status and prognosis. Together, these markers offer a comprehensive approach to diagnosing HB, monitoring treatment response, and predicting clinical outcomes. Their integration into routine clinical practice could significantly improve patient stratification and personalized treatment plans.

### 3.3. Imaging Techniques

HB diagnosis relies on advanced imaging techniques such as US, computed tomography (CT), and magnetic resonance imaging (MRI), complemented by specialized methods like digital subtraction angiography (DSA), positron emission tomography (PET), and 3D Visual Analysis. US is commonly used as the first imaging modality to detect liver lesions, distinguishing solid and cystic components and monitoring changes during treatment. CT scans provide detailed information about the tumor’s extent, size, and vascular involvement and are essential for assessing metastasis, particularly through chest CT, which has shown 97% accuracy in detecting lung metastasis in children with HB [97]. CT is also instrumental in staging according to the PRETEXT system, revealing multifocal lesions and guiding treatment decisions. MRI, especially diffusion-weighted imaging (DW-MRI), is considered the gold standard in HB imaging, as it can detect satellite lesions missed by CT, offering a more accurate staging of the disease [98]. Furthermore, MRI plays a crucial role in assessing vascular involvement, including portal and hepatic venous issues, which are essential for treatment planning.

In cases with complex presentations, such as those with hepatic arteriovenous fistulas (HAVF), DSA provides a detailed view of the tumor’s vascular structures, aiding in surgical and interventional planning. PET scans are used to assess metabolic activity and detect metastasis, particularly in cases where conventional imaging may fall short. PET scans can help differentiate between benign and malignant lesions, providing additional insight into the tumor’s biological behavior [99]. For pediatric patients with large or strategically positioned tumors, 3D Visual Analysis using enhanced CT or MRI provides a more accurate preoperative assessment. This technique is especially beneficial for complex tumors, allowing for more precise surgical planning and minimizing potential risks during resection [100]. These imaging modalities, together with clinical evaluation, ensure a comprehensive approach to diagnosing and managing HB.

### 3.4. Diagnostic Path

The diagnostic path for HB involves the integration of serum biomarkers, IHC markers, and advanced imaging techniques to ensure accurate diagnosis, staging, and treatment planning. The process typically begins with serum biomarkers, with AFP being the most widely used for assessing tumor burden and recurrence. The AFP-L3 fraction is particularly useful for predicting recurrence after surgery [91]. Additionally, ctDNA, specifically CTNNB1 mutations, is a sensitive marker for tumor burden and treatment response [92], while SUA serves as a promising prognostic tool, correlating with advanced tumor stages and poor treatment responses [93]. Following serum biomarker testing, IHC markers help to further characterize the tumor, with β-catenin expression indicating poor chemotherapy response and increased vascular involvement [94]. GS is used to assess tumor differentiation, especially post-chemotherapy [95], and CD203c is associated with more aggressive tumor phenotypes, aiding in prognosis [96]. To finalize the diagnosis and staging, advanced imaging techniques are employed. US is typically the first imaging modality for detecting liver lesions and monitoring treatment, while CT provides detailed information on tumor size, extent, and vascular involvement, especially for staging and metastasis evaluation [97]. MRI, particularly DW-MRI, is the gold standard for detecting satellite lesions and assessing vascular involvement [98]. In more complex cases, DSA provides a detailed view of the tumor’s vascular structures, and PET helps evaluate metabolic activity and metastasis [99]. For large or complex tumors, 3D Visual Analysis with enhanced CT or MRI offers a more accurate preoperative assessment, improving surgical planning [100]. This comprehensive diagnostic approach, combining biomarkers, IHC markers, and advanced imaging, ensures a personalized and effective treatment plan for patients with HB. Table 3 summarizes the systematic comparisons between the various diagnostic methods.

## 4. Treatment of Hepatoblastoma (HB)

The treatment of HB requires a multidisciplinary approach incorporating chemotherapy, surgery, immunotherapy, and targeted therapies. The primary goals are to achieve complete tumor resection, control metastasis, and overcome chemotherapy resistance, aimed at improving survival rates, particularly in advanced cases. Each therapeutic modality targets distinct aspects of tumor biology to achieve optimal outcomes.

### 4.1. Chemotherapy

Chemotherapy plays a critical role in the treatment of HB, particularly in advanced or metastatic cases, with the aim of reducing tumor size, controlling metastasis, and enhancing resectability to improve survival rates. Neoadjuvant chemotherapy is essential for shrinking tumors prior to surgery. HB chemotherapy regimens have evolved significantly from the late 20th century to the early 21st century, with two primary international research directions: the International Society of Paediatric Oncology Liver Tumour Study Group (SIOPEL) and Children’s Oncology Group (COG). The SIOPEL 1 trial (1990–1994) first introduced preoperative chemotherapy using a combination of CDDP and doxorubicin (DOX) in the PLADO regimen, improving the OS rate to 57%, although it showed limited efficacy in patients with metastatic disease [101,102]. Subsequent studies, such as SIOPEL 2 (1995–1998), introduced risk stratification, categorizing patients into standard risk (SR) and high-risk (HR) groups, with SR patients achieving a 3-year OS of 91% [103]. SIOPEL 3 (1998–2004) further optimized treatment for HR patients by introducing alternating cycles of CDDP and carboplatin (CARBO) with DOX, resulting in a 3-year OS of 69% [104]. SIOPEL 4 (2005–2009) improved the 3-year OS for HR patients to 83% with intensified chemotherapy, although it was associated with increased side effects [105]. The COG study (2009–2012) focused on the C5VD regimen (CDDP + 5-FU + VCR + DOX), achieving a 5-year OS of 95% in patients with unresectable HB [106].

Tumor rupture, considered a high-risk feature in SIOPEL 3/4 regimens, was associated with poor prognosis, indicating risks of peritoneal progression or recurrence. However, it should not be considered an absolute contraindication for LT [107]. In resource-limited countries, studies have shown that CDDP monotherapy remains the optimal treatment for SR patients, even outperforming the PLADO regimen in survival rates [108]. In Japan, the intensified CDDP regimen in SIOPEL-4 was well tolerated and effective for HR patients [109]. Additionally, novel therapies such as Panobinostat, a MYC protein inhibitor, have shown promising experimental efficacy against metastatic HB. When combined with CDDP and DOX, Panobinostat enhances treatment efficacy, offering new potential for improving outcomes in HB treatment [110]. Chemotherapy remains a vital component for both advanced and congenital HB, with ongoing research into personalized therapies showing promise for the future. Table 4 presents the chemotherapy for HB. The drugs mentioned in the treatment regimens, including CDDP and DOX, are considered first-line treatments due to their established efficacy in various international protocols such as PLADO, C5VD, and SIOPEL studies. CARBO, which is often used in combination with CDDP, is generally considered a second-line option, particularly for high-risk patients who require intensified therapy. Similarly, 5-FU, VCR, and Panobinostat, which are used in the C5VD regimen and in combination with CDDP and DOX, are generally categorized as second-line drugs. Panobinostat, in particular, is still under investigation and is not yet standard therapy.

### 4.2. Surgical Treatment

Surgical treatment is crucial in managing HB, aiming for complete tumor resection to improve survival. The choice between liver resection and liver transplantation (LT) depends on tumor location, vascular involvement, and resectability at diagnosis. Liver resection is preferred for resectable HB, but LT may be required for those with major vascular involvement or multifocal tumors. Uchida et al. [111] found that children with tumor thrombus extending into major vascular structures, such as the portal vein or hepatic veins, often require LT, with good survival outcomes for portal vein involvement.

In congenital HB, neoadjuvant chemotherapy is key for making resection possible. Li et al. [100] highlighted a case where chemotherapy followed by 3D-guided surgery resulted in complete remission. Extreme liver resection after chemotherapy, demonstrated by Xiu et al. [112], offers a viable alternative to LT. Minimally invasive techniques, including indocyanine green (ICG) fluorescence imaging and 3D simulation systems, are improving surgical precision. Studies by Qiu et al. [113] and Liu et al. [114] showed that ICG enhances tumor boundary identification during surgery, while 3D planning improves liver function preservation. These advances have reduced the reliance on LT and improved surgical outcomes.

### 4.3. Immunotherapy

The use of immune checkpoint inhibitors (ICIs) and macrophage-targeting therapies is emerging as a promising strategy in the treatment of HB, particularly for patients with refractory or metastatic disease. One case study demonstrated the efficacy of pembrolizumab, an ICI, in a patient with PRETEXT III HB who developed recurrent lung metastases despite multiple treatments. The patient achieved a complete and sustained response for 22 months, emphasizing the potential of ICIs, particularly for those with a high tumor mutation burden (TMB) [115]. Additionally, GPC3, a protein overexpressed in HB, is being targeted by immunotherapies such as vaccines, monoclonal antibodies, and Chimeric Antigen Receptor T-cell therapy (CAR T-cells) [116]. However, GPC3 is physiologically expressed in the liver and kidneys during early life, which necessitates careful consideration of potential immune-related side effects in young children [16]. Meanwhile, TAMs, which support tumor progression and immune evasion, are also being targeted in HB. The reprogramming of TAMs, through macrophage ICIs and chimeric antigen receptor (CAR) macrophage therapies, aims to restore their anti-tumor function, potentially enhancing the body’s immune response to HB and overcoming its resistance to conventional therapies [87].

In addition to immune-based strategies, pyroptosis induction is gaining attention as a potential therapeutic approach for enhancing cancer treatment. Pyroptosis, a form of programmed cell death associated with inflammation, promotes the release of pro-inflammatory cytokines, triggering a strong immune response against tumor cells. Physical therapies such as radiotherapy, sonodynamic therapy, and photodynamic therapy can induce pyroptosis in cancer cells, which may increase their susceptibility to ICIs and other immune-based treatments. This approach has the potential to work synergistically with ICIs by stimulating immune cells, such as NK cells and cytotoxic T lymphocytes (CTLs), to attack cancer cells [117]. Additionally, midkine, an immune-modulating molecule upregulated in HB, could be targeted to enhance the anti-tumor immune response further [89]. By combining pyroptosis induction with immune therapies, it is possible to overcome the immune evasion mechanisms in HB, improving the overall therapeutic efficacy. These combined approaches offer a promising strategy for treating HB, especially in cases resistant to conventional therapies.

Immunotherapy, particularly the use of ICIs, has shown promise in treating HB, but it is also associated with immune-related adverse events (irAEs). These adverse events arise from excessive immune activation and can affect various organs, complicating treatment. Common irAEs associated with ICIs include hepatitis, pneumonitis, thyroid dysfunction, skin disorders, and renal dysfunction [118,119]. Hepatic dysfunction is particularly frequent and, in severe cases, can lead to acute liver failure, while pneumonitis can manifest as interstitial pneumonia, worsening prognosis if untreated. Endocrine disturbances, such as hypothyroidism, are also commonly observed with pembrolizumab therapies and may be linked to better OS in certain cancers like hepatocellular carcinoma [120]. Additionally, ICIs can cause severe coagulation disorders, including acquired coagulation factor deficiency, leading to life-threatening bleeding, and immune thrombocytopenia (ITP), especially with nivolumab and pembrolizumab [121,122]. These adverse events highlight the importance of careful monitoring and early intervention, often with corticosteroids or other immunosuppressive treatments, to manage severe irAEs [123]. While ICIs offer promising therapeutic potential for HB, clinicians must remain vigilant to the risks associated with these therapies and adjust treatment protocols as necessary.

### 4.4. Targeted Therapy

Recent advances in targeted therapies for HB offer new treatment options, particularly for chemoresistant and high-risk cases. Key strategies focus on inhibiting molecular pathways involved in tumor growth, metastasis, and chemotherapy resistance. For example, PIM kinase inhibition using AZD1208, combined with CDDP, has shown synergy in reducing tumor cell proliferation and inducing cell cycle arrest in metastatic HB by targeting the ATM DNA damage response pathway [124]. Similarly, panobinostat, a histone deacetylase (HDAC) inhibitor, combined with VCR and irinotecan (CPT-11), has demonstrated significant tumor regression in preclinical models of treatment-refractory HB, highlighting its potential for use in high-risk cases [125]. Another promising approach involves dapagliflozin, a Sodium-Glucose Cotransporter 2 (SGLT2) inhibitor, which, when combined with CDDP, has been shown to reduce CDDP resistance in HB cells by inhibiting glucose uptake, thus restoring CDDP sensitivity [126]. Inosine Monophosphate Dehydrogenase 2 (IMPDH2) inhibition with mycophenolate mofetil (MMF), combined with DOX, has also proven effective in enhancing therapeutic responses in HB by inducing cell cycle arrest and apoptosis [127]. Furthermore, mebendazole, an anthelmintic drug, combined with CDDP, showed a synergistic effect in overcoming CDDP resistance, significantly reducing tumor growth and inducing apoptosis without notable side effects in preclinical models [128]. Finally, the cellular inhibitor of apoptosis protein 1 (cIAP1) inhibition using birinapant, combined with CDDP, enhanced tumor suppression by overcoming the resistance to apoptosis in CDDP-resistant HB cells [129]. These therapies, still largely in clinical trials or preclinical stages, offer promising strategies to improve outcomes for HB patients, particularly those with chemotherapy-resistant disease, and may provide significant advancements in the treatment of high-risk HB.

In conclusion, the treatment of HB has evolved with significant advancements across chemotherapy, surgery, immunotherapy, and targeted therapies. Ongoing research continues to improve survival and quality of life for children with HB, with personalized approaches offering hope for better outcomes. Table 5 summarizes the treatment methods and strategic plans for managing HB.

## 5. Conclusions

HB remains a significant clinical challenge due to its aggressive nature, particularly in high-risk and metastatic cases. Despite advancements in molecular understanding, diagnostic techniques, and treatment modalities, significant obstacles such as chemotherapy resistance, tumor recurrence, and treatment-related toxicity persist. Current therapies, including chemotherapy, surgery, immunotherapy, and targeted treatments, address various aspects of HB biology. However, their efficacy varies across patient subgroups, underscoring the need for more individualized approaches. A promising direction for the future is the integration of Traditional Chinese Medicine (TCM) remedies, which may improve outcomes by enhancing immunity and alleviating the side effects of conventional treatments. For example, Ginsenoside Rh1, a compound from ginseng, has shown potential in regulating the immune microenvironment by promoting immune activation, which could enhance the effectiveness of conventional therapies like lenvatinib. Additionally, G-Rh1 has been shown to modulate the glucocorticoid receptor (GR) and increase the infiltration of immune cells such as CD8+ T cells, dendritic cells, and MHC-I-positive cells, making it a valuable adjunct for treating HB and improving immune responses [130].

Looking ahead, personalized medicine that integrates genetic and epigenetic profiling is essential for stratifying HB patients based on risk and treatment response. The development of novel biomarkers, such as ctDNA and specific ncRNAs, will significantly enhance early diagnosis, prognosis, and disease monitoring. Additionally, the application of machine learning models and radiomics in imaging analyses offers exciting possibilities for precise staging and surgical planning. A deeper understanding of key signaling pathways in HB, including Wnt/β-catenin, Hippo, and PI3K/Akt/mTOR, reveals promising therapeutic targets. Future research should focus on developing targeted therapies and combination treatments that effectively disrupt these pathways while minimizing toxicity. The exploration of immunotherapies, such as CAR-T cell therapies and macrophage modulation, holds great potential for addressing chemoresistant and advanced HB cases. Furthermore, the continued advancement of epigenetic drugs and small-molecule inhibitors will broaden the spectrum of treatment options. A multidisciplinary approach that integrates advances in molecular biology, bioinformatics, and clinical medicine is crucial to address the complexity of HB. Ultimately, these efforts aim to improve survival rates, reduce treatment-related side effects, and enhance the quality of life for children affected by HB.

## Figures and Tables

**Table 1:** The role and mechanisms of lncRNAs and miRNAs in hepatoblastoma (HB).

lncRNAs/miRNAs	Regulatory Axis or Signaling Pathway	Effect on Tumor	References
Linc00205	miR-154-3p/ROCK1 axis	Promotes tumorigenesis	[34]
	MAPK pathway	Promotes tumorigenesis	[34]
MIR205HG	MAPK pathway	Promotes tumorigenesis	[35]
	PI3K/AKT pathway	Promotes tumorigenesis	[35]
Linc01023	miR-378a-5p/WNT3 axis	Promotes tumorigenesis	[36]
HAND2-AS1	HAND2-AS1/CDK1 axis	Inhibits tumorigenesis	[37]
NEAT1	miR-132/MMP9 axis	Promotes tumorigenesis	[38]
miR-135a	Notch pathway	Inhibits tumorigenesis	[39]
miR-139-3p	miR-139-3p/Wnt5A axis	Inhibits tumorigenesis	[40]

Abbreviations: lncRNAs, long non-coding RNAs; miRNAs, microRNAs.

**Table 2:** The signaling pathways involved in hepatoblastoma (HB) and their regulatory roles in tumorigenesis.

Signaling Pathway	Genes in the Pathway	Effect on Tumors	References
Wnt/β-Catenin pathway	β-Catenin, Fascin-1, KDM1A	Promotes tumorigenesis	[50,51,52]
	SOX7	Inhibits tumorigenesis	
Hippo pathway	YAP, TAZ, VEGF	Promotes tumorigenesis	[56,57,58,59]
Notch pathway	Notch2	Promotes tumorigenesis	[62]
TGF-β pathway	TGF-β1, Smad2/3, Smad4	Promotes tumorigenesis	[65]
IGF pathway	IGF2, IGF1R	Promotes tumorigenesis	[69,70,71]
PI3K/AKT/mTOR pathway	mTORC1, DPEP1	Promotes tumorigenesis	[73,74]
MAPK/ERK pathway	POSTN, ERK,	Promotes tumorigenesis	[77,78]
HGF/c-MET pathway	c-Met, RALY	Promotes tumorigenesis	[80,81]

Abbreviations: TGF-β, transforming growth factor-beta; IGF, insulin-like growth factor; HGF, hepatocyte growth factor; POSTN, periostin; YAP, yes-associated protein 1; VEGF, vascular endothelial growth factor; TAZ, transcription regulator 1.

**Table 3:** Systematic comparison of diagnostic methods.

Diagnostic Method	Key Features	Advantages	Limitations	References
Serum Markers	AFP: Key marker for diagnosis and monitoring of HB.	Non-invasive, easy to measure, provides initial insights into tumor burden, recurrence, and prognosis.	AFP is less specific for HB vs. other liver conditions.	[91]
	AFP-L3: Sensitive for predicting recurrence following surgery.	More sensitive than AFP for predicting recurrence.	Limited availability, requires specialized assays.	[91]
	ctDNA: Sensitive for detecting tumor mutations (CTNNB1).	Non-invasive, highly sensitive, correlates with tumor burden, treatment response, and progression.	Requires specialized sequencing techniques.	[92]
	SUA: Prognostic marker linked to advanced tumor stages and poor treatment responses.	Provides insight into tumor staging and predicts clinical outcomes.	SUA’s predictive value is still emerging, and its role is not fully established.	[93]
IHC Markers	β-catenin: Key in HB pathogenesis and a strong prognostic indicator.	Provides prognostic insights regarding chemotherapy response, vascular involvement, and survival.	Requires biopsy and tissue sample; not useful for real-time monitoring.	[94]
	GS: Assesses tumor differentiation and detects residual disease.	Useful for evaluating differentiation, especially post-chemotherapy, and for detecting residual disease.	Requires biopsy and tissue sample; may not be available in all settings.	[95]
	CD203c: Indicator of aggressiveness, especially in less differentiated tumors.	Helps identify more aggressive tumor phenotypes, aiding in prognosis and treatment planning.	Requires biopsy; limited availability in clinical practice.	[96]
Imaging Techniques	US: Initial detection of liver lesions, distinguishing solid and cystic components.	Non-invasive, accessible, cost-effective, useful for monitoring during treatment.	Limited in resolution and detail for staging and vascular involvement.	[97]
	CT: Provides detailed anatomical imaging, including tumor size, extent, and staging.	Offers comprehensive anatomical details, crucial for tumor staging and metastasis evaluation.	Radiation exposure, misses smaller lesions compared to MRI, less sensitive for vascular assessment.	[97]
	MRI: Gold standard for accurate staging, satellite lesions detection, and vascular involvement.	High resolution, especially with DW-MRI, providing detailed staging and vascular assessment.	Expensive, less accessible, requires specialized equipment.	[98]
	DSA: Evaluates tumor vasculature, especially in cases with HAVF.	Provides a detailed view of the tumor’s vascular structures, aiding surgical and interventional planning.	Invasive, requires contrast agents, and is used only in specific cases.	[99]
	PET: Assesses metabolic activity, especially in metastatic cases.	Helps detect metastasis and assesses tumor metabolic behavior, especially when conventional imaging is insufficient.	Expensive, radiation exposure, and limited availability in some settings.	[99]
	3D Visual Analysis: Enhances preoperative assessment, particularly for complex or large tumors.	Improves surgical planning by providing precise, three-dimensional models of the tumor, especially useful for complex tumors.	Requires advanced imaging technologies (CT/MRI), may not be available in all centers.	[100]

Abbreviations: IHC, immunohistochemistry; AFP, alpha-fetoprotein; SUA, serum uric acid; ctDNA, circulating tumor DNA; US, ultrasound; CT, computed tomography; MRI, magnetic resonance imaging; DW-MRI, diffusion-weighted imaging; DSA, digital subtraction angiography; PET, positron emission tomography; GS, glutamine synthetase.

**Table 4:** The chemotherapy regimens for hepatoblastoma (HB).

Study Period	Study Protocol	Chemotherapy Regimen	First-Line or Second-Line	Efficacy	Applicability	References
1990–1994	SIOPEL 1	PLADO (CDDP + DOX)	First-line	5-year OS: 57%	Approved	[101,102]
1995–1998	SIOPEL 2	CDDP (SR)/PLADO (HR)	First-line	SR-3-year OS: 91%; HR-3-year OS: 53%	Approved	[103]
1998–2004	SIOPEL 3	CDDP + CARBO + DOXO (HR)	First-line	3-year OS: 69%	Approved	[104]
2005–2009	SIOPEL 4	Dose-dense CDDP + CARBO + DOX (HR)	First-line	3-year OS: 83%	Approved	[105]
2009–2012	COG-C5VD	C5VD (CDDP + 5-FU + VCR + DOX) (MR)	First-line	5-year OS: 95%	Approved	[106]
2000–2014	SIOPEL 3/4	CDDP + CARBO + DOX (HR)	First-line	3-year OS: 68.2%	Approved	[107]
2021	JPLT3-H	Dose-dense CDDP + CARBO + DOX (HR)	First-line	-	Approved	[109]
2022	SIOPEL	CDDP monotherapy vs. PLADO	First-line	SR group: CDDP monotherapy superior to PLADO	Approved	[108]
2024	Panobinostat + SIOPEL 4	Panobinostat + CDDP + DOX	Not yet established	Combination therapy shows efficacy	Pre-clinical trials	[110]

Abbreviations: CDDP, cisplatin; CARBO, carboplatin; DOX, doxorubicin; 5-FU, 5-fluorouracil; VCR, vincristine; HR, high risk; SR, standard risk; MR, middle risk; OS, overall survival; SIOPEL, International Society of Paediatric Oncology Liver Tumour Study Group; COG, Children’s Oncology Group.

**Table 5:** The treatment methods for hepatoblastoma (HB).

Treatment Methods	Treatment Plans	Treatment Mechanism	Applicability	References
Surgical treatment	Liver resection	Resectable HB	approved	[111]
	LT	Cases with major vascular involvement or multifocal tumors	approved	[111]
	Minimally invasive techniques, including ICG fluorescence imaging and 3D simulation	Improved surgical precision and reduced LT reliance	clinical trials	[100,112,113]
Immunotherapy	Pembrolizumab	Block immune checkpoint proteins (e.g., PD-1/PD-L1), enhancing the immune system’s ability to attack tumor cells	approved	[110]
	GPC3-targeted vaccines, monoclonal antibodies and CAR-T cell therapies	Activated immune system targets GPC3-expressing tumor cells	clinical trials	[16,111]
	Macrophage immune checkpoint inhibitors and CAR macrophage therapies	Reprograms TAMs to enhance immune response	clinical trials	[112]
Targeted therapy	AZD1208 (PIM inhibitor) + CDDP	Target tumor cell proliferation in metastatic HB by inhibiting the ATM DNA damage response pathway	pre-clinical trials	[115]
	Panobinostat (HDAC inhibitor) + VCR + CPT-11	Restore normal gene expression and overcome chemotherapy resistance	pre-clinical trials	[116]
	Dapagliflozin (SGLT2 inhibitors) + CDDP	Overcome CDDP resistance by reducing glucose uptake in tumor cells	pre-clinical trials	[117]
	IMPDH2 inhibitors + DOX	Inhibiting IMPDH2 enhances the effectiveness of chemotherapy agents like DOX	pre-clinical trials	[118]
	Mebendazole + CDDP	Inhibit tumor growth, especially in chemoresistant HB	pre-clinical trials	[119]
	Birinapant (cIAP1 inhibitors) + CDDP	Overcome resistance to CDDP by inhibiting the proteins that block apoptosis in HB cells	pre-clinical trials	[120]

Abbreviations: HB, hepatoblastoma; LT, liver transplantation; GPC3, Glypican 3; ICG, indocyanine green; CAR-T, chimeric antigen receptor T-cell; HDAC, histone deacetylase; SGLT2, Sodium-Glucose Cotransporter 2; IMPDH2, Inosine Monophosphate Dehydrogenase 2; cIAP1, cellular inhibitor of apoptosis protein 1; CDDP, cisplatin; DOX, doxorubicin; VCR, vincristine; CPT-11, irinotecan.
